# An Approach to the Use of Depth Cameras for Weed Volume Estimation

**DOI:** 10.3390/s16070972

**Published:** 2016-06-25

**Authors:** Dionisio Andújar, José Dorado, César Fernández-Quintanilla, Angela Ribeiro

**Affiliations:** 1 Center for Automation and Robotics, Spanish National Research Council, CSIC-UPM, Arganda del Rey, Madrid 28500, Spain; angela.ribeiro@csic.es; 2 Institute of Agricultural Sciences, Spanish National Research Council, CSIC, Madrid 28006, Spain; jose.dorado@ica.csic.es (J.D.); cesar@ica.csic.es (C.F.-Q.)

**Keywords:** Kinect v2, weed/crop structure characterization, weed detection, plant volume estimation, maize

## Abstract

The use of depth cameras in precision agriculture is increasing day by day. This type of sensor has been used for the plant structure characterization of several crops. However, the discrimination of small plants, such as weeds, is still a challenge within agricultural fields. Improvements in the new Microsoft Kinect v2 sensor can capture the details of plants. The use of a dual methodology using height selection and RGB (Red, Green, Blue) segmentation can separate crops, weeds, and soil. This paper explores the possibilities of this sensor by using Kinect Fusion algorithms to reconstruct 3D point clouds of weed-infested maize crops under real field conditions. The processed models showed good consistency among the 3D depth images and soil measurements obtained from the actual structural parameters. Maize plants were identified in the samples by height selection of the connected faces and showed a correlation of 0.77 with maize biomass. The lower height of the weeds made RGB recognition necessary to separate them from the soil microrelief of the samples, achieving a good correlation of 0.83 with weed biomass. In addition, weed density showed good correlation with volumetric measurements. The canonical discriminant analysis showed promising results for classification into monocots and dictos. These results suggest that estimating volume using the Kinect methodology can be a highly accurate method for crop status determination and weed detection. It offers several possibilities for the automation of agricultural processes by the construction of a new system integrating these sensors and the development of algorithms to properly process the information provided by them.

## 1. Introduction

New trends in agriculture allow for the precise management of the spatial occurrence of pests within agricultural fields. The requirement of agriculture to have a low impact on the environment and the challenge of feeding an increasing population provides precision agriculture (PA) with the opportunity to face this challenge. PA uses knowledge of spatial and temporal variations in crops. The management of the spatio-temporal information needs methods and technologies that improve day by day to fulfill new requirements of food sustainability processes that optimize available resources [1]. Pests significantly curtail agricultural production and are responsible for a decrease of approximately 40% in potential global crop yields because they transmit disease, feed on crops, and compete with crop plants [2]. A major issue in Europe is the current reliance on chemical methods of pest control (CE 1107/2009 and 2009/128/CE). Thus, site-specific crop management could be the solution for a lower environmental impact while maximizing yields. The case of weed management is of high importance because herbicides are the most used pesticides in the world. The introduction of the concept of site-specific weed management (SSWM) is an attempt to manage the heterogeneity of fields though the use of new sensors and machinery to precisely treat weed patches only, so that the use of herbicides can be drastically reduced. The use of SSWM is conditioned by crop value, the proportion of the field infested by weeds, the shape and number of the patches, and the technologies for sampling and spraying [3]. The use of sensing technologies can separate weed from crops, identifying patches within the field through weed characteristics such as color or height. The quantification of these values is needed to determine correct management. Young et al. [4] noted a 53% reduction in the applications of grass herbicides in wheat through using SSWM. Similarly, Gerhards and Christensen [5] showed a herbicide reduction greater than 75% in grass weeds in a wheat crop over four years. Andújar et al. [6] put forward that SSWM was the most profitable strategy with normal levels of infestation and showed herbicide savings of more than 80% in maize crops. High-value crops such as horticultural plants or fruit trees, where only a small proportion of the field is infested by weeds, are the ideal targets for SSWM because the benefits can highly justify the associated cost of detection [7]. The increasing adoption of SSWM techniques is driven by the increasing knowledge of new farmers and the related economic benefits of using new technologies. In addition, some directives of the European Union lead to a reduction of the inputs used for pest control (European Council 2009, Brussels, Belgium), indirectly promoting the use of precision agriculture technologies, which allow for the reduction of pesticide use.

Currently, new tools for managing the heterogeneity within agricultural fields are emerging from new sensors and combined systems. When applied to crops, these systems allow cost optimization by new cost-efficient management and minimization of the environmental impact of tillage and chemical products. They are mainly based on different types of sensors that are able to discriminate weeds and crops or reconstruct plant shapes by phenotyping methods. Phenotyping techniques characterize plant structure by using non-invasive methodologies with new sensors that have been recently developed. The geometrical characterization of plant structures leads to an understanding of internal processes and establishes differences among plant species for good identification. The evaluation and modeling of plant architecture using phenotyping requires high precision and accuracy for measuring protocols [8]. An accurate model improves the decisions about the use of the information taken by phenotyping. Plant phenotyping models are created though different types of sensors, from imaging to non-imaging techniques, that recreate plant shapes [9]. Different systems can be used for this purpose. Weed identification using machine vision by portable imaging and analysis software is the most investigated technique [10]. However, under outdoor agricultural environments, different problems appear. The problem of variable and uncontrolled illumination that, among other things, produces shadows and leaf overlapping is a major challenge [11]. Spectral reflectance sensors [12,13] and fluorescence sensors [14] are also used. Regarding distance sensors, ultrasonic sensors and LiDAR are sensors available on the market. These sensors can be used for plant height and volume estimation [14,15]. In addition, other systems have been explored for plant characterization, including radar systems, stereovision, magnetic resonance, and thermal imaging. 

Structured-light scanners have opened a new door in 3D modeling. They are automatic 3D acquisition devices that create high fidelity models of real 3D objects in a highly time-effective way and at low cost. There are two major manufacturers in the market: Microsoft Kinect 1.0 and 2.0 (Microsoft, Redmond, WA, USA) and the Asus Xtion (Asus, Taipei, Taiwan). Asus Xtion and Kinect 1.0 sensors combine structure light with computer vision techniques: depth from focus and depth from stereo [16]. Chéné et al. [17] reconstructed the geometric shape of rosebush, yucca, and apple tree and introduced an automatic algorithm for leaf segmentation from a single top view image acquisition. The algorithm could be applied to automate processes for plant disease or stress detection because leaf morphology can be related to internal processes in the plant. Wang & Li [18] applied the RGB-depth Kinect sensor to estimate onion fruit diameter and volume. The comparison between the measurements taken with RGB images and those taken with RGB-D showed a higher average accuracy for RGB-D. Plant structure characterization is a widely known technique that uses similar sensors based on time-of-flight (ToF) cameras [19,20], and is used for the estimation of the foliar density of fruit trees to control a spraying system [21]. Wang et al. [22] developed a picking robot that imitated human behavior for fruit recollection. Agrawal et al. [23] designed an inexpensive robotic system that was affordable for growers and was compatible with the existing hydroponic infrastructure for growing crops such as lettuce. Paulus et al. [24] compared a low-cost 3D imaging system with a high-precision close-up laser scanner for phenotyping purposes. The measured parameters from the volumetric structure of sugar beet taproots, the leaves of sugar beet plants, and the shape of wheat ears were compared. Although the study showed myriad possibilities for using depth cameras in plant phenotyping and the potential of using these sensors in automated application procedures, their reliability was lower than that of laser scanners. However, the low cost and additional information provided by RGB makes it a good alternative to high-cost tools. Although light radiation impedes its use in outdoor experiments, the use of the new Kinect v2 sensor allows for its use under high illumination conditions because the measurement principle is based on ToF methodology [25,26]. It also has a higher resolution capacity and can process a higher volume of data with a wider field of view. This paper presents a novel solution to separate weeds and maize plants through depth images taken in real outdoor conditions of maize fields, demonstrating its capabilities and limitations in discriminating weeds from crops using color segmentation and plant height measurements.

## 2. Material and Methods

### 2.1. Data Collection System

The Kinect v2 sensor has a depth camera, an RGB camera of 1080p, and an infrared emitter. The depth sensor is based on an indirect ToF measurement principle. An infrared light is emitted and reflected by the impacted object. The time it takes for light to travel from the infrared illuminator to the impacted object and back to the infrared camera is stored and transformed by wave modulation and phase detection to calculate the distance between the emitter and the object. The RGB camera has a resolution of 1920 × 1080 pixels, giving it an array of pixel RGB values that need to be converted into a WPF representation. The IR (infrared) camera used for the acquisition of depth data, as the depth camera, has a resolution of 512 × 424 pixels that allows the sensor to work in darkness and allows for the tracking of IR reflective objects while filtering out IR lights. When sensing depths of 70 degrees horizontally and 60 degrees vertically, the sensor can take in information from a wider field of view than the previous version of the sensor. Thus, objects can be closer to the sensor and still in its field of view, and the camera is also effective at longer distances, covering a larger total area. Although the technical characteristics provided by Microsoft state that the operative measurement range works from 0.5 m to 4.5 m, some tools enable the reconstruction of bigger 3D meshes by using ICP (Iterative Closest Point) algorithms, by moving the Kinect sensor around the scanned object. These meshes can be processed and edited as unstructured 3D triangular meshes in different software applications. The sensor readings are composed of a high number of frames per second with overlapping areas in the continuous measurements. The overlapped zones allow for the creation of better models and the automatic removal of outliers in the mesh. The software merges the points into a common point cloud taken by the relative position of the sensor, which is relative to itself at the initial point.

**Figure 1:**
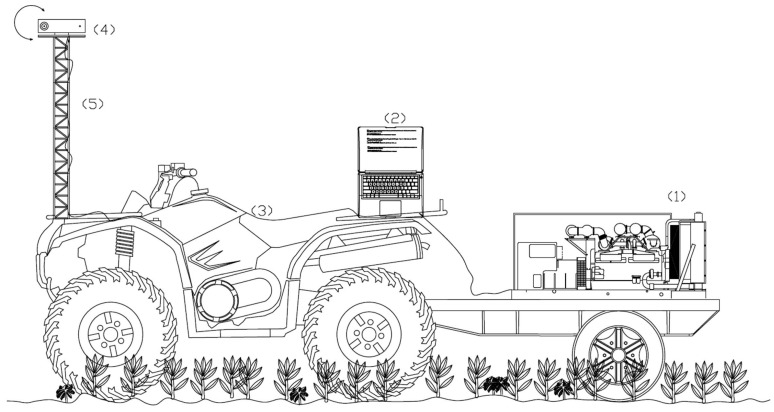
Schematic design of the system with the components integrated for maize-weed detection. (1) Portable gasoline generator; (2) laptop; (3) ATV; (4) Kinect v2 sensor; (5) support structure.

Meshes were acquired from the Kinect v2 sensor running Software Development Kit (SDK) on an Intel desktop computer with Windows 8 (Microsoft, Redmond, WA, USA). The data acquisition software was developed starting from the official Microsoft SDK 2.0 (Microsoft, Redmond, WA, USA)), which includes the drivers for the Kinect sensor. It allows the necessary drivers for the sensor to be obtained, as well as the use of a set of customizable sample functions that were implemented for the measurements, combined with some OpenCV functions. The aim was to store both depth and RGB information at a rate of at least 25 frames per second. The data were processed using a volumetric reconstruction based on a memory and speed efficient data structure [27]. The chosen storage format was compatible with the open software (Meshlab^®^, University of Pisa, Pisa, Italy) that was subsequently used to process the acquired raw data. The system was supplied with electric power by a portable gasoline generator of 6 kw. The Kinect sensor and additional devices were mounted in an ATV (All Terrain Vehicle, Yamaha, Shizuoka, Japan). The Kinect sensor was positioned in the middle of the sampling area and the ATV was stopped. Then, measurements were taken for 1 to 2 s, moving the sensor mechanically with a rotator motor from 45° to −45°, avoiding the effect of leaf overlapping by displacing the sensor through different point of views (Figure 1).

### 2.2. Field Measurements

**Figure 2:**
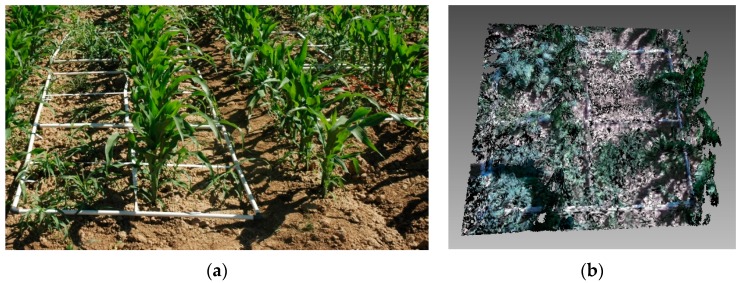
(**a**) Some frames located in the experimental field; (**b**) example of a mesh obtained with the Kinect v2 sensor.

The experimental set up was conducted under field conditions at La Poveda Research Farm (Arganda del Rey, Madrid, Spain). The soil was conventionally tilled. Maize was planted on the 6 April 2015 with 0.75-m row spacing and a population of 90,000 plants/ha. The experimental field was infested with *Sorghum halepense* (L.) Pers., *Datura ferox* L., *Salsola kali* L., *Polygonum aviculare* L., and *Xanthium strumarium* L., with some other minor weeds. Weeds were always shorter than maize plants. Weed control treatments were based on pre-emergence and post-emergence herbicide applications. Weed assessments were made in May when maize was at stage 14 to 16 of BBCH scale [28] and weeds BBCH 12 to BBCH 22 [29] (Figure 2).

The sample locations were chosen to search for different weed compositions of grass, broad-leaved weeds and crops, as well as mixtures of them. The samples were from pure samples of the different species, as well as an equitable distribution of different mixtures and compositions. Weeds and crops were sampled using an 1 m × 1 m frame, which was divided into four quadrats. A total of ten 1 m × 1 m frames were sampled. Then, samples were distributed in quadrats of 0.5 × 0.5 m^2^, with a total of 40 samples. Half of the quadrats were positioned in the inter-row area, and half of them were located in the maize row. Thus, the samples were equally distributed in areas with crops and areas occupied only by weeds. Some of the samples were weed-free or maize-free due to their positions. Because weed detection needs to be fast due to the narrow window for weed treatment, a full 3D model for an entire field would not be realistic. Thus, single 3D models were constructed from the top position. Every reading was taken in direct sunlight under real field conditions at midday on a sunny day (average of 40,000 lux) and not using any shading structure. Following the readings, the density and biomass of the weeds and maize were assessed within the quadrats (0.5 m × 0.5 m) located in the row and inter-row positions. Weed emergences were counted in each sample quadrat by species. Plants were cut and processed in the lab to determine the dry weights of the different species.

### 2.3. Data Processing

**Figure 3:**
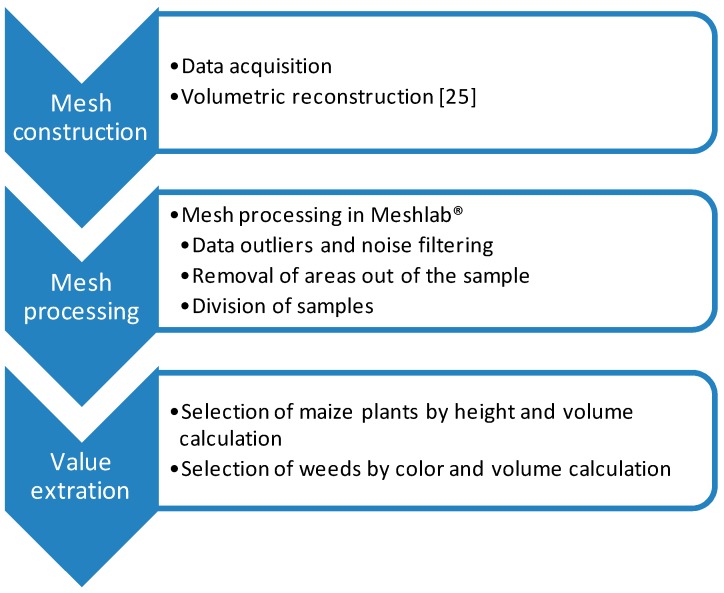
Data processing structure of the Kinect sensor information.

**Figure 4:**
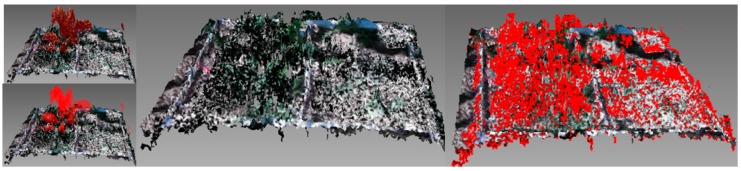
An example of maize isolation and removal by height selection, and an example of color selection for weed extraction.

The meshes were processed to calculate maize and weed volume (Figure 2b). The offline processing of the samples was conducted using the open software Meshlab^®^ (3D-CoForm, Brighton, UK). The initial point cloud was processed in three steps [30,31] (Figure 3): (a) Data outliers and noise in the point cloud were filtered out. The filter identifies the points with no connection (1 cm out of the grid) and removes them. (b) The areas out of the 1 m × 1 m frame were removed and the mesh was divided into samples of 0.5 m × 0.5 m. The division and removal of areas out of the frame were automatically done based on the color segmentation of the white frame. (c) Plant volumes were calculated from the mesh by computation of polyhedral mass properties [32]. Firstly, the maize parts of the mesh were isolated by height (Figure 4). Because the maize plants were considerably taller than the weeds, the upper part and the connected faces of the mesh were selected. From this selection, a new mesh was created, and maize volume was extracted. Then, after the selection and removal of the maize plants in the mesh, an RGB filter was applied to select the reaming green parts corresponding to weeds. The mesh volume of weeds was then obtained. The calculated volumes extracted from the meshes were statistically compared with the actual weight values, which were manually assessed.

### 2.4. Statistical Analysis

The data of maize and weed volume obtained with Kinect v2 sensor were analyzed and compared with the actual values of dry biomass weight using regression methods. Pearson’s correlation coefficients were calculated to evaluate the simple linear relationships between the actual parameters and those obtained with the sensor. A correlation analysis was made prior to the regression analysis to provide initial information regarding the bivariate relationships among the parameters.

A canonical discriminant analysis (CDA) was used to predict weed classification of the system using weed height. This methodology is based on pattern recognition to find an optimal linear combination of features for the separation into groups. The dependent variable is expressed as functions of linear combinations of the input features [33]. The method tried to show the capabilities of the method to separate (a) infested samples by broad-leaved weeds; (b) infested samples by grasses; and (c) infested by mixture of both weed types. The analyses were performed using the SPSS statistical software (v. 20, IBM Corporation, Armonk, NY, USA).

## 3. Results and Discussion

**Figure 5:**
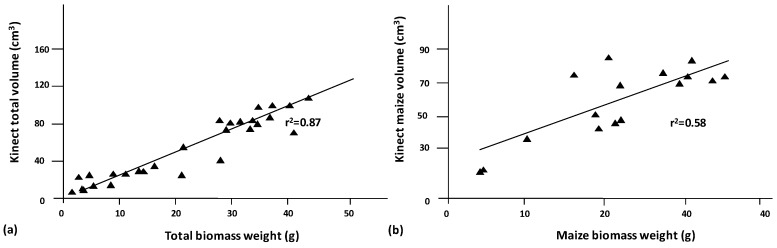
Linear regression between both the total volume that was estimated using the Kinect v2 sensor and the total biomass (**a**) and the maize volume with the maize biomass (**b**). The *R*^2^ denotes the correlation coefficient of the simple regression.

The constructed models obtained by scanning maize samples using the new Kinect v2 device showed a high correlation for the calculated structural parameters between maize and weed soil measurements. The maize plants that were present in the sample always demonstrated a greater height than the weeds, which allowed for the identification of maize plants in the sample. Maize plants ranged from 40 cm to 55 cm, while weeds varied depending on weed species. *Sorhgum halepense* was the highest weed with a maximum height of 20 cm, while broad-leaf weeds were always shorter than 10 cm. The height of maize plants allowed for the selection of the upper part of the mesh and the connected faces of the mesh for the separation of maize and weeds. In addition, the difference in height between grasses and broad-leaf weeds identified both types of weeds. Maize plants had a higher volume in the sample than weeds. The shorter height of weeds led to a lower volume and necessitated RGB recognition to separate them from the microrelief of the samples. High correlations between the constructed models and the ground-truth variables resulted in significance at a level of 1%. The total volume of the model was highly correlated with the total vegetation weight, with an *r* of 0.93. Maize volume was well correlated with maize weight, with a Pearson’s *r* of 0.77. The correlation between weed weight and weed volume reached an *r*-value of 0.83. These values show the potential of the new Kinect sensor in crop identification and weed detection. Figure 5a shows the linear regression for total biomass and total volume, with a high relation between values. In addition, once the maize volume was isolated from the sample, the maize biomass weight and maize volume showed a good relationship (Figure 5b). Thus, Kinect v2 volume estimation is an accurate method for crop status determination. This information could be useful for fertilizer applications using the same principles but with other sensors such as NVDI, ultrasonic, and LiDAR sensors. Thus, those areas with a higher or lower volume could receive a differential dose of fertilizers according to the growing state. Similar studies have shown the capability of Kinect sensors to characterize plant volume for several uses. The authors agree that the characterization of plant structures with Kinect leads to an overestimation of the actual plant values because the sensors cannot reconstruct the end details. Nock et al. [34] could not reconstruct *Salix* branches below 6.5 mm in diameter using an Asus Xtion, whereas a Kinect v1 worked with branches ranging from 2 to 13 mm in diameter at a distance of 0.5–1.25 m from the target object. However, they agree that these low-cost 3D imaging devices for plant phenotyping are reliable and can be integrated into automated applications of agricultural tasks [24]. Although the measurements tend to overestimate the actual dimensions of the plants, as in our experiments, plant reconstruction is of high fidelity compared with RGB images, and 3D modeling using this sensor constitutes a highly valuable method for plant phenotyping. In addition, the method worked properly at very early weed stages, which is when most of the weed treatments should be applied. Thus, this technology may be applicable for weed identification at various crop growth stages. Although some other methodologies, such as LiDAR, measure distance with a higher resolution, its inability to reproduce RGB limits the capability for weed and soil separation.

**Figure 6:**
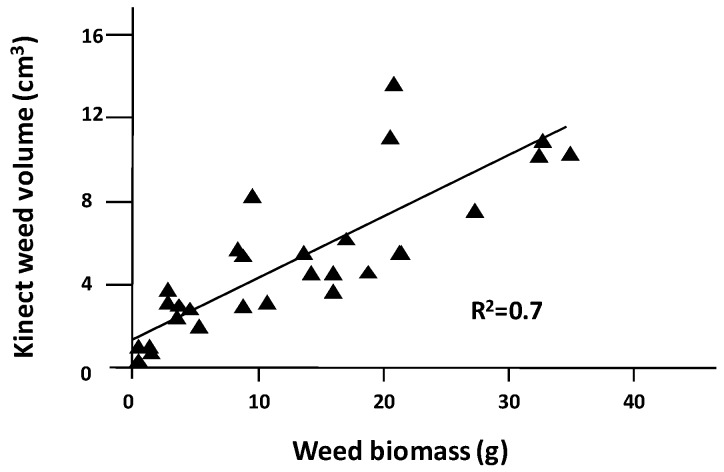
Simple linear regression between the total weed volume that was calculated with the depth and color images and the weed biomass weight.

In addition, the discrimination of the color of the weeds opens a new window for weed control. The Kinect v1 sensor had a lower resolution and an inability to work in outdoor conditions due to lighting conditions, which were the main deterrents to its usage. The improvements in the Kinect v2 sensor allowed for the acquisition of meshes outdoors with real and uncontrolled lighting. The separation of weed areas from the mesh and the calculated volume resulted in a linear correlation between the volumes estimated from the models and the actual parameters of weed biomass. The regression showed an *r*^2^ of 0.7, indicating a good relationship with the volume data obtained with the Kinect device. Figure 6 shows the simple regression for weed biomass and weed volume, with a good relation between values, which demonstrate the suitability of the sensor for weed detection. Although RGB recognition is a good complement for weed isolation in 3D models, using color-image processing by itself for volume calculation results in worse predictions than depth images [18].

When the plant densities were analyzed, the number of maize plants did not show a significant correlation with the calculated model. This fact was mainly due to the almost constant number of maize plants per sample. On the contrary, weed density showed good correlations with the volumetric measurements. They resulted in a significant difference: *p* < 0.01. The total volume of the model was correlated with weed density, with an *R*^2^ of 0.59. Once the weed volume was isolated from the rest of sample elements, a similar *R*^2^ value of 0.6 was obtained.

The CDA showed promising results for classification into three predefined groups (Table 1). All of the samples infested exclusively by broad-leaf weeds were properly classified (100%). In addition, 92.3% of the cases infested with presence of grasses were classified correctly. More than a half (53.8%) were properly classified as pure samples of grass weeds. The rest of the cases (38.5%) were classified as mixture of grasses and broad-leaf grasses. Samples composed of mixtures were classified as mixtures and grasses in the same proportion. Incorrect classification of mixtures could be due to the fact that the maximum height obtained is based on grasses present in the sample. Although the method did not allow for the discrimination of grasses and mixture, this does not suppose a major practical problem if a mixture of herbicides would be used on SSWM when a higher height is detected.

The system has shown large potential to work in outdoor conditions. Although further studies in software development are necessary to improve the results of the data processing stage, the sensor shows several potential applications that could not be considered for the previous version of Kinect. The sensor is able to work under natural high-lighting conditions, collecting a high number of points with accurate resolution. RGB detection, which is also improved, allowed for the separation of weeds from soil in the sample, creating an opportunity for real-time weed control applications. The possible applications of 3D models open a new door to continue the development and optimization of precision agriculture processes. Although the use of 3D models for geometric characterization of vegetation is not new, the sensors fulfill some needs regarding processing time, robustness, and information quality to adapt to the requirements of real-time agricultural applications. 

Similar to this study, Chen et al. [35] derived some structural parameters of maize using the Kinect v1 version. The authors stated that leaf area index and leaf angle can be properly described with this sensor. Thus, considering this information and the plant volume extracted in the current study, the sensor can help to manage the information coming from the field to gain a better understanding of plant growth. Previous studies have shown that structure and size calculations from meshes are similar to manual measurements [31]. Concerning the use of RBG information, the camera in the Kinect v2 sensor has been improved and is in fact even better at identifying weeds in the samples. Yamamoto et al. [36] described a procedure for acquiring plant information on strawberry plants. The information on height, width, volume, leaf area, leaf inclination, and leaf color was assessed using a Kinect v1 sensor and compared with the actual results. The results agreed well with experimental evidence, and similar values of volume agreement to those of the current study were shown. In addition, motion information allowed for the segmentation of videos, avoiding the effect of shadows and overlap, one of the major problems in 2D imaging for weed classification. Thus, the impact of depth cameras on new trends in SSWM is high. The potential possibilities of greater automation of agricultural processes should be studied and improved by the construction of a new system integrating these sensors. The development of better algorithms for information processing will also be necessary. These innovations can increase productivity and reduce herbicide use within agricultural fields using the current sensing technology that is able to detect weeds where they are present, combined with an automatic system to treat weeds site-specifically.

## 4. Conclusions

The possibilities and disadvantages of depth sensors regarding their practical use in weed detection were assessed based on results obtained from field experiments. The dual methodology using height selection and RGB segmentation properly separated crops, weeds, and soil. Depth data provided geometrical information to the model and allowed for the discrimination of maize plants due to their higher height compared with weeds. A similar methodology could be used in other row crops, where crops have different heights compared with weeds or where weeds are located out of the crop row. In addition, the separation between broad leaf weeds and grasses was achievable using the same principle as height selection. The RGB data provided enough information for the separation of vegetation from soil. Thus, depth data provided geometrical information for classifying features according to their location and RGB according to its color. The constructed models show the potential of depth sensors to collect and fuse the spatial and color information of crops and to extract information for several purposes. The application of selective treatments using the proposed methodology would allow for a higher reduction of herbicide use in maize fields. In addition, the low cost and high frame rate makes this sensor a promising tool for site-specific weed management.

The proposed system offers opportunities for automation of agricultural processes by integrating depth cameras with fast algorithms. The possibilities for on-the-go operations and the development of new algorithms to automatically and more quickly build 3D models need to be further explored. These sensors and algorithms need to be used and integrated to properly process the captured information.

## Figures and Tables

**Table 1:** Confusion matrix of the canonical discriminant classification showing a percentage of correct group classifications for the three predefined groups.

		Predicted	
	Monocots	Dicots	Mixture
Monocots	53.8	7.7	38.5
Dicots	0.0	100.0	0.0
Mixture	50.0	0.0	50.0
